# Comprehensive analysis of suppressor of cytokine signaling proteins in human breast Cancer

**DOI:** 10.1186/s12885-021-08434-y

**Published:** 2021-06-13

**Authors:** Mingyu Sun, Chuangang Tang, Jun Liu, Wenli Jiang, Haifeng Yu, Fang Dong, Caiguo Huang, Youlutuziayi Rixiati

**Affiliations:** 1 Department of Breast Surgery, Xuzhou Central Hospital, The Affiliated Xuzhou Hospital of Medical College of Southeast University, Xuzhou, 221009 China; 2 Department of Biochemistry and Molecular Biology, College of Basic Medical, Navy Medical University, Shanghai, 200433 China; 3 Department of General Surgery, Tianjin First Central Hospital, Tianjin, 300192 China; 4 Department of Vascular Surgery, Gansu Provincial Hospital, Lanzhou, 730000 China; 5 Department of Pathology, Soochow University Medical School, Suzhou, 215123 China

**Keywords:** Breast cancer, Bioinformatics, Prognosis, Treatment, SOCS3

## Abstract

**Background:**

Abnormal expression of suppressor of cytokine signaling (SOCS) proteins regulates tumor angiogenesis and development in cancers. In this study, we aimed to perform a comprehensive bioinformatic analysis of SOCS proteins in breast invasive carcinoma (BRCA).

**Methods:**

The gene expression, methylation level, copy number, protein expression and patient survival data related to SOCS family members in BRCA patients were obtained from the following databases: Oncomine, The Cancer Genome Atlas (TCGA), Genotype-Tissue Expression (GTEx), Human Protein Atlas (HPA), Gene Expression Profiling Interactive Analysis (GEPIA), PCViz, cBioPortal and Kaplan-Meier plotter. Correlation analyses, identification of interacting genes and construction of regulatory networks were performed by functional and pathway enrichment analyses, weighted gene coexpression network analysis (WGCNA) and gene set enrichment analysis (GSEA).

**Results:**

Data related to 1109 BRCA tissues and 113 normal breast tissue samples were extracted from the TCGA database. SOCS2 and SOCS3 exhibited significantly lower mRNA expression levels in BRCA tissues than in normal tissues. BRCA patients with high mRNA levels of SOCS3 (*p* < 0.01) and SOCS4 (*p* < 0.05) were predicted to have significantly longer overall survival (OS) times. Multivariate analysis showed that SOCS3 was an independent prognostic factor for OS. High mRNA expression levels of SOCS2 (*p* < 0.001), SOCS3 (p < 0.001), and SOCS4 (*p* < 0.01), and a low expression level of SOCS5 (p < 0.001) were predicted to be significantly associated with better recurrence-free survival (RFS). Multivariate analysis showed that SOCS2 was an independent prognostic factor for RFS. Lower expression levels of SOCS2 and SOCS3 were observed in patients with tumors of more advanced clinical stage (*p* < 0.05). Functional and pathway enrichment analyses, together with WGCNA and GSEA, showed that SOCS3 and its interacting genes were significantly involved in the JAK-STAT signaling pathway, suggesting that JAK-STAT signaling might play a critical role in BRCA angiogenesis and development. Western blot results showed that overexpression of SOCS3 inhibited the activity of the JAK-STAT signaling pathway in vitro.

**Conclusions:**

SOCS family proteins play a very important role in BRCA. SOCS3 may be a prognostic factor and SOCS2 may be a potential therapeutic target in breast cancer.

**Supplementary Information:**

The online version contains supplementary material available at 10.1186/s12885-021-08434-y.

## Background

Breast cancer is one of the most common malignant tumors in women and has a very high incidence, accounting for 25% of all cancer cases in women worldwide [1, 2]. The currently established clinical treatments for breast cancer mainly include surgery, radiotherapy, and chemotherapy [3]. However, the considerably high rates of recurrence and metastasis of breast cancer make the effects of its clinical treatment unsatisfactory, leading to generally poor patient prognosis [4, 5]. Therefore, it is important to identify alternative targets for establishing individualized treatment of patients with breast cancer and to develop novel biomarkers to enhance the prognosis of these patients.

The suppressor of cytokine signaling (SOCS) family is a family of immunosuppressive proteins that was recently discovered and contains 8 members, including cytokine-inducible SH2-containing protein (CIS) and SOCS 1–7 proteins [6, 7]. Since cytokine signaling plays an essential role in the initiation and development of human cancer, various studies have focused on elucidating the relationships between the expression of SOCS family members and different cancer types [8, 9]. It has been reported that the abnormal expression of SOCS proteins can regulate cancer development in various tumor cell types, as well as in immune cells in the tumor microenvironment [10].

SOCS proteins, including SOCS1, 2 and 3, act as negative regulators of the prolactin pathway in the mammary epithelium [11]. To date, only a few studies have reported the role of SOCS genes in breast cancer. Sutherland et al. demonstrated that SOCS1 and SOCS2 can inhibit the growth of breast cancer cells [12]. Using fresh frozen breast cancer tissue samples (*n* = 127) and normal breast tissues (*n* = 31), Sasi et al. evaluated the expression levels of SOCS 1–7, and found higher mRNA expression levels of SOCS1, 3, 4, and 7 were significantly associated with early-stage tumor and more favorable prognosis in human breast cancer patients [13]. A recent study suggested that SOCS1 expression was low in breast cancer tissues and its expression level was associated with different clinical stages of breast cancer [6]. However, the distinctive roles of SOCS family proteins in breast cancer and the underlying mechanisms by which they are derepressed or activated have not been fully elucidated.

Therefore, using data published online from The Cancer Genome Atlas (TCGA) and the Genotype-Tissue Expression (GTEx) databases, we aimed to determine the expression patterns, potential functions and unique prognostic value of SOCS proteins in breast invasive carcinoma (BRCA).

## Methods

### Date extraction from Oncomine database

The gene expression microarray data were downloaded from Oncomine database, which is an online database of different cancer microarray experiments [14]. Oncomine microarray datasets were used to analyze the expression levels of the SOCS family in different human cancers. The mRNA expression levels of the SOCS family in multiple tumor samples were compared with those in normal samples. The thresholds for determining statistical significance were set as *p* < 0.05 and fold change (FC) > 1.5.

### Date extraction from GEPIA database

Gene Expression Profiling Interactive Analysis (GEPIA) is a novel interactive web-based application, which was used for analyzing the gene expression datasets based on 9736 tumor and 8587 normal samples extracted from TCGA and the GTEx databases [15]. Using the GEPIA extracted datasets, the mRNA expression levels of SOCS family members between BRCA and normal tissues were compared. Additionally, the relationship between the mRNA expression levels of the SOCS family and different tumor stages of BRCA was also analyzed.

### Survival analysis using Kaplan-Meier plotter database

Kaplan-Meier plotter is an online database, which has gene expression and survival data including BRCA patients [16]. The prognosis for BRCA patients, in terms of overall survival (OS) and recurrence-free survival (RFS), was analyzed using the Kaplan-Meier plotter. Briefly, according to the median of the mRNA expression levels of SOCS genes, the patient samples were divided into two groups (high vs. low expression). Thereafter, the OS and RFS of the BRCA patients were compared between the high and low expression groups. Multivariate Cox proportional hazard regression analysis of OS and RFS were performed to determine independent prognostic factors.

### cBioPortal online tool

TCGA database has the pathological and sequencing data of 30 different human cancers [17]. BRCA genomic profiles were selected from TCGA (Provisional) for analyzing alterations in the SOCS family by using the cBioPortal online tool.

### Immunohistochemistry images from the human protein atlas

The Human Protein Atlas (HPA) is the best resource for the discovery of proteins and biomarkers, as it includes more than 5 million images of immunohistochemically stained cells and tissues. In this study, we downloaded the immunohistochemistry images of SOCS proteins in breast cancer cells. The staining pattern was defined as negative, low, medium or high.

### Date extraction from the TCGA database

The RNA-seq data (Level 3, Counts) were downloaded using R package (v.3.6.1) and TCGA biolinks v2.14. The ENSEMBL ID in RNA-seq was re-annotated using the GTF annotations file in GENCODE v.32 [18] to extract the corresponding gene symbols. The mRNAs were extracted based on the annotation information. Since multiple ENSEMBL IDs may correspond to the same gene symbol, their average was taken as the unique expression value corresponding to the mRNA of interest.

### Co-expression analysis

The read counts in the expression profile were subjected to log2 transformation. The gene expression values of SOCS family members (SOCS1–7) in BRCA samples were extracted. The co-expression patterns between every two SOCS genes were analyzed using the Pearson’s correlation coefficient method. The expression correlation diagram was drawn using the R package corrplot v.0.84 (https://github.com/taiyun/corrplot).

### SOCS genes regulatory network analysis

The regulatory networks of SOCS family members and their interacting genes was constructed using the PCViz tool (http://www.pathwaycommons.org/pcviz/). The interaction types included “state change”, “gene expression” and “in the same protein complex”. The 50 most relevant interacting genes were selected to build the final regulatory networks.

### Functional enrichment analysis

The genes identified in the network were subjected to Kyoto Encyclopedia of Genes and Genomes (KEGG) and Gene Ontology (GO) (biological process (BP), molecular function (MF) and cellular component (CC)) enrichment analyses using the clusterProfiler v3.14.0 [19]. The KEGG pathway and GO terms with the screening thresholds of *p*-value < 0.05 were selected, and the top 10 KEGG pathways and GO terms are shown as bubble chart.

### Weighted gene Coexpression network analysis and gene set enrichment analysis

Weighted gene coexpression network analysis (WGCNA) was conducted to investigate the co-expressed genes of SOCS3 using R WGCNA package. (https://cran.r-project.org/package=WGCNA). The minimum module size was set as 30. Gene set enrichment analysis (GSEA) using default parameters were performed to explore the potential pathways where SOCS3 might be involved (http://www.broad.mit.edu/gsea).

### Timer 2.0

TIMER 2.0 (http://timer.cistrome.org/) was used to investigate the association between the expression level of SOCS3 and immune infiltration. This tool provides several accesses for assessment of the immune infiltration including TIMER, EPIC, CIBERSORT and etc. EPIC results were selected.

### MEXPRESS

MEXPRESS is a database for visualizing the relationship between gene expression, methylation and clinical information of patients in TCGA database (https://mexpress.be/). Common clinical parameters for BRCA, including ER status, PR status, HER2 status, gender and stage, were selected for further analysis of SOCS3.

### Overexpression of SOCS3

The CDs fragment of SOCS3 gene was connected to pIRES2 plasmid after digested by EcoR I and BamH I. Then pIRES2-SOCS2 plasmid was transformed to DH5α competent cells. Next, screening by kanamycin was performed. Finally, the pIRES2-SOCS2 plasmid was extracted, sequenced and compared with the gene bank, while pIRES2-NC was as control.

### Western blot

Cells were lysed in radioimmunoprecipitation assay (RIPA) buffer and proteins were extracted. BCA protein assay kit was used to determine the concentrations of protein samples. Then protein samples were separated by 10% SDS-PAGE and transferred to NC membrane. Next, the membrane was blocked and incubated in primary antibody at 4°Cfor 8 h (p-JAK, JAK1, p-STAT3, STAT3, SOCS3, and β-actin at 1:1000, 1:1000, 1:1000, 1:1000, 1:1000, and 1:5000, respectively). Then the membrane was incubated in secondary antibody (1:10,000) for 90 min after washed by TBST for four times. At last, the protein expression was detected by ECL chemiluminescence buffer and tested by an Odyssey v3.0 image scanner (LI-COR Biosciences, NE, USA). Band was analyzed by Image J software.

## Results

### Expression of SOCS family proteins in multiple cancers

**Fig. 1:**
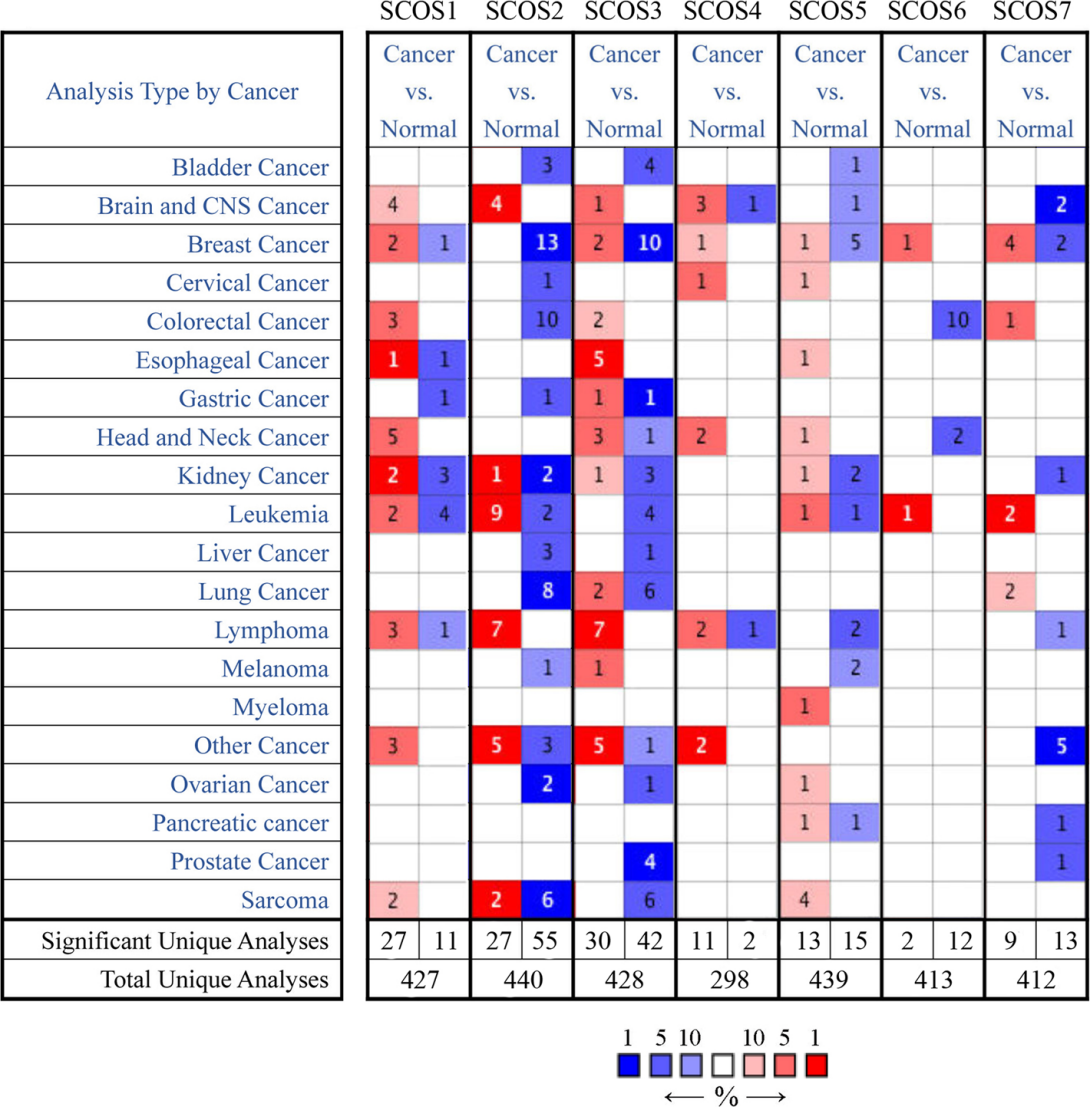
mRNA expression levels of SOCS family members in different types of cancer (Oncomine). Cell color is determined by the best gene rank percentile for the analyses within the cell. The numbers mean the number of analyses which meet our threshold

The expression levels of SOCS family proteins in human cancer samples compared with normal tissue samples were analyzed using the Oncomine database (Fig. 1). Regarding SOCS1 expression, a total of 427 studies of multiple cancer types were considered, among which 38 studies showed statistical differences. Regarding SOCS2 expression, a total of 440 studies were considered, among which 27 showed increased expression and 55 showed decreased expression in tumor tissues compared with normal tissues. Regarding SOCS3 expression, 428 studies were considered, among which 30 showed increased expression and 42 showed decreased expression in tumor tissues compared with normal tissues. A total of 298 studies were considered for analysis of SOCS4 expression, among which 11 showed increased expression and 2 showed decreased expression in tumor tissues compared with normal tissues. A total of 439 studies were considered for analysis of SOCS4 expression, among which 13 showed increased expression and 15 showed decreased expression in tumor tissues compared with normal tissues. For SOCS5 and SOCS6, 413 and 412 studies of multiple cancer types were taken into consideration, respectively. However, 14 and 22 of these studies showed statistically significant differences in SOCS5 and SOCS6 expression, respectively, between tumor and normal tissues.

Only three studies on BRCA were found that showed statistically significant differences in SOCS1 expression: two showed increased expression, and one showed decreased expression in tumor tissues compared with normal tissues. Additionally, 13 studies were identified that reported significantly decreased expression of SOCS2 in BRCA. Two studies reporting significantly increased expression and 10 reporting significantly decreased expression in tumor tissues were identified for SOCS3. For SOCS4 and SOCS6, only one study reporting increased expression in tumor tissues was identified. For SOCS5, one study reporting increased expression and five studies reporting decreased expression in tumor tissues were identified. Furthermore, for SOCS7, four studies reporting increased expression and two reporting decreased expression in tumor tissues were identified.

### Expression of SOCS family members in patients with BRCA

**Fig. 2:**
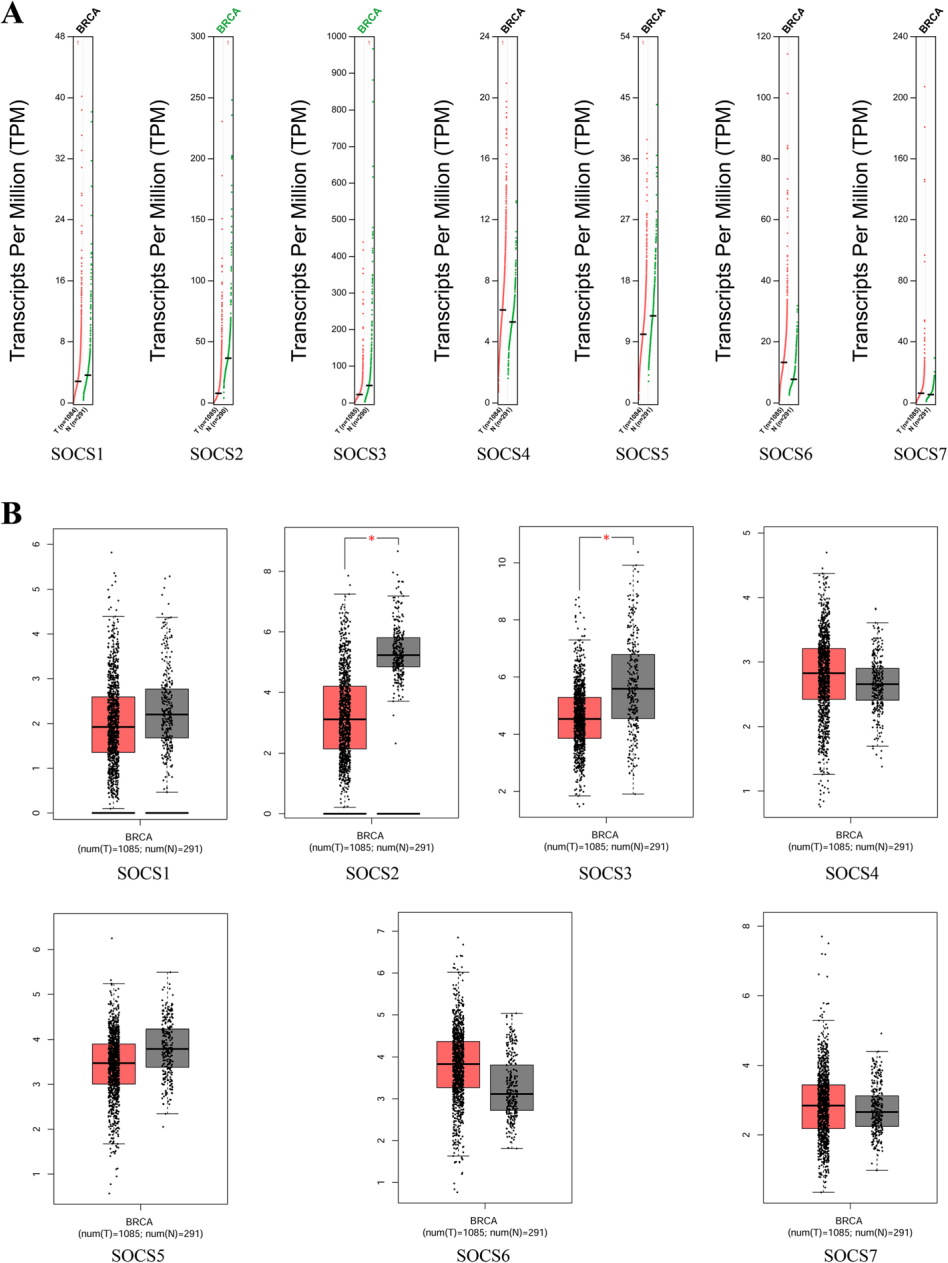
mRNA expression levels of SOCS family members in BRCA (GEPIA). (**a**) Scatter diagram. **b** Box plot. Grey color represents the normal tissue and red color represents the tumor tissue. * denotes *p* < 0.05

The mRNA expression of SOCS family members in tumor and normal tissues was compared by GEPIA dataset analysis. As shown in **Fig.** **2****A** and **B**, the expression levels of SOCS2 and SOCS3 were significantly lower in BRCA than in normal breast tissues. Oncomine database analysis, as shown in Table 1, indicated significant differences in SOCS mRNA expression levels between tissues of different subtypes of breast cancer and normal breast tissues. With the thresholds of *p*-value< 0.05, fold change≥2, and gene rank = top 10%, SOCS1 was determined to be overexpressed in invasive breast carcinoma and invasive lobular breast carcinoma tissues compared with normal tissues based on data from TCGA; SOCS2 was determined to be down-regulated in breast carcinoma based on data from TCGA, Ma et al. [20], Curtis et al. [21], Karnoub et al. [22], Turashvili et al. [23], Gluck et al. [24] and Richardson et al. [25]; and SOCS3, SOCS4, SOCS5, SOCS6 and SOCS7 were determined to be highly expressed in breast carcinoma based on data from TCGA and Ma et al. [20], Finak et al. [26], Turashvili et al. [23], Radvanyi et al. [27].

**Table 1:** The Significant Change of SOCS Expression in Transcription Level between Different Types of Breast Cancer and Normal Breast Tissues

SOCS	Type of Breast Cancer versus Normal Breast Tissue	Fold Change	***P*** Value	T Test	Source and/or Reference
SOCS1	Invasive Lobular Breast Carcinoma	2.010	1.92E-10	7.108	TCGA
	Invasive Breast Carcinoma	2.076	1.19E-13	8.166	TCGA
SOCS2	Invasive Ductal Breast Carcinoma Stroma	−2.710	1.27E-6	− 6.825	Ma et al.
	Ductal Breast Carcinoma in Situ Epithelia	−2.203	0.001	−3.456	Ma et al.
	Invasive Ductal Breast Carcinoma	−5.707	1.52E-46	−20.589	TCGA
	Invasive Breast Carcinoma	− 4.347	3.49E-21	− 11.498	TCGA
	Intraductal Cribriform Breast Adenocarcinoma	−8.398	0.008	−6.596	TCGA
	Invasive Ductal Breast Carcinoma	−3.286	2.40E-82	−29.894	Curtis et al.
	Medullary Breast Carcinoma	−2.479	4.50e-19	−13.918	Curtis et al.
	Invasive Breast Carcinoma	−2.925	8.58E-7	−6.462	Curtis et al.
	Breast Carcinoma	−3.007	3.18E-5	−5.634	Curtis et al.
	Invasive Ductal Breast Carcinoma Stroma	−2.611	5.76E-4	−4.293	Karnoub et al.
	Invasive Lobular Breast Carcinoma	−3.527	0.035	−2.344	Turashvili et al.
	Invasive Breast Carcinoma	−4.560	0.002	−6.624	Gluck et al.
	Ductal Breast Carcinoma	−6.257	1.14E-4	−5.440	Richardson et al.
SOCS3	Invasive Ductal Breast Carcinoma Epithelia	2.554	1.20E-4	4.474	Ma et al.
	Invasive Breast Carcinoma Stroma	3.056	8.40E-16	11.078	Finak et al.
SOCS4	Invasive Ductal Breast Carcinoma	2.019	0.037	1.898	Turashvili et al.
SOCS5	Invasive Ductal Breast Carcinoma Epithelia	2.239	0.005	3.109	Ma et al.
SOCS6	Invasive Lobular Breast Carcinoma	3.099	0.016	2.593	Radvanyi et al.
SOCS7	Invasive Ductal Breast Carcinoma	2.415	1.85E-30	15.856	TCGA
	Intraductal Cribriform Breast Adenocarcinoma	2.599	4.85E-4	9.365	TCGA
	Invasive Breast Carcinoma	2.047	5.37E-14	8.349	TCGA
	Mucinous Breast Carcinoma	2.029	0.006	4.436	TCGA

**Fig. 3:**
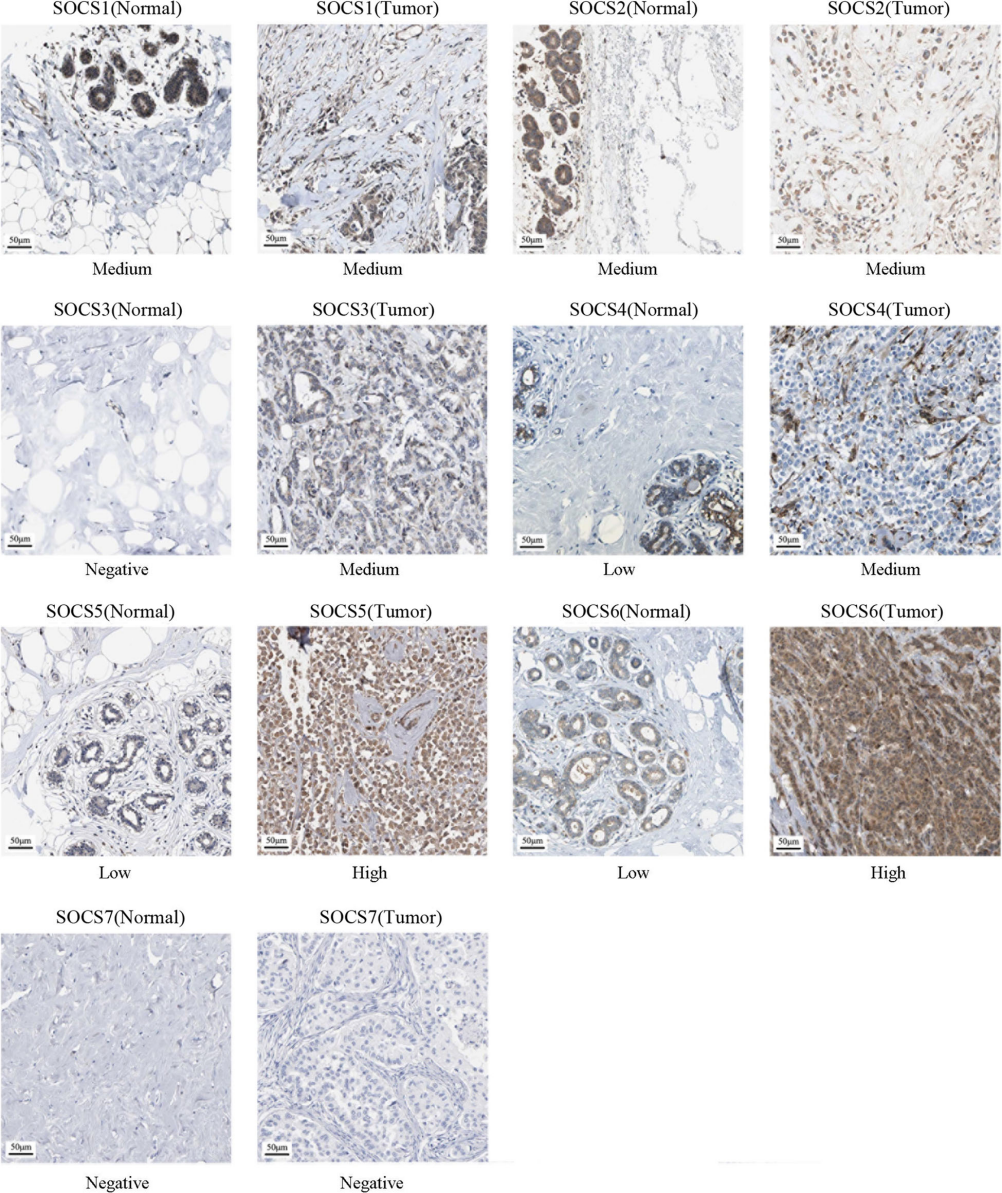
Protein expression levels of SOCS family members in BRCA tumor tissues and normal tissues based on immunohistochemistry images (HPA)

In addition to the mRNA expression levels, we also assessed the protein expression levels of SOCS family members based on the immunohistochemical images of SOCS proteins in BRCA patients obtained from the HPA database. As shown in Fig. 3, SOCS2 had lower expression in BRCA tissues than in normal tissues (antibody staining level: high vs. medium), consistent with the mRNA expression pattern of SOCS2. However, the HPA provided only one image of SOCS3 protein staining in normal breast tissues and no antibody staining was detected. As a result, the protein expression levels of SOCS3 in BRCA tissues and normal tissues could not be statistically compared.

### Effects of SOCS family members on the prognosis of BRCA patients

**Fig. 4:**
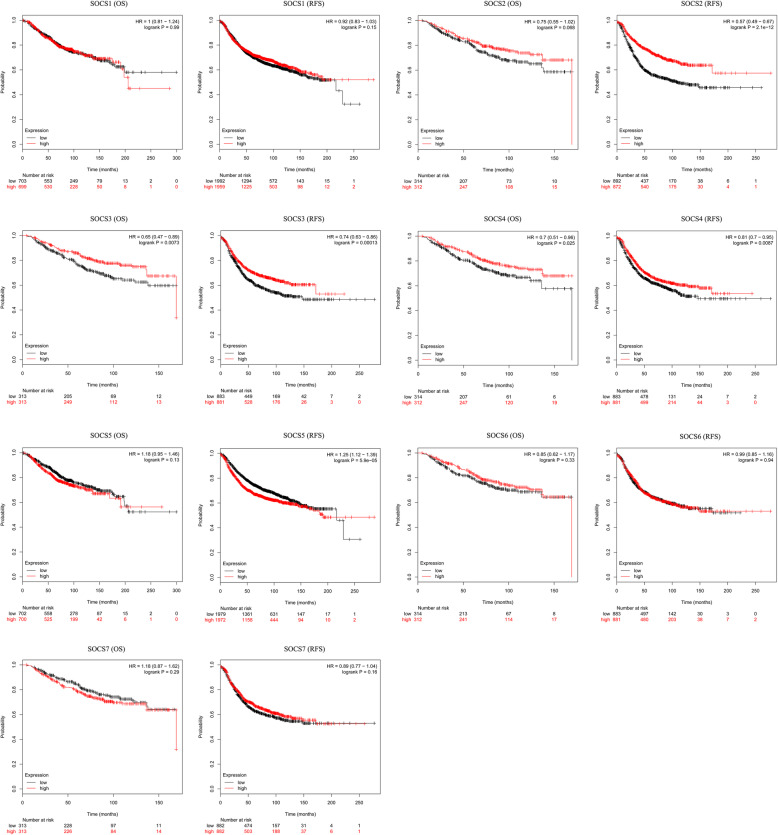
Prognostic values of SOCS family members in BRCA patients (Kaplan-Meier plotter). BRCA patients are stratified into high-expression and low-expression groups according to the median expression levels of SOCS family members. p < 0.05 is considered statistically significant

**Fig. 5:**
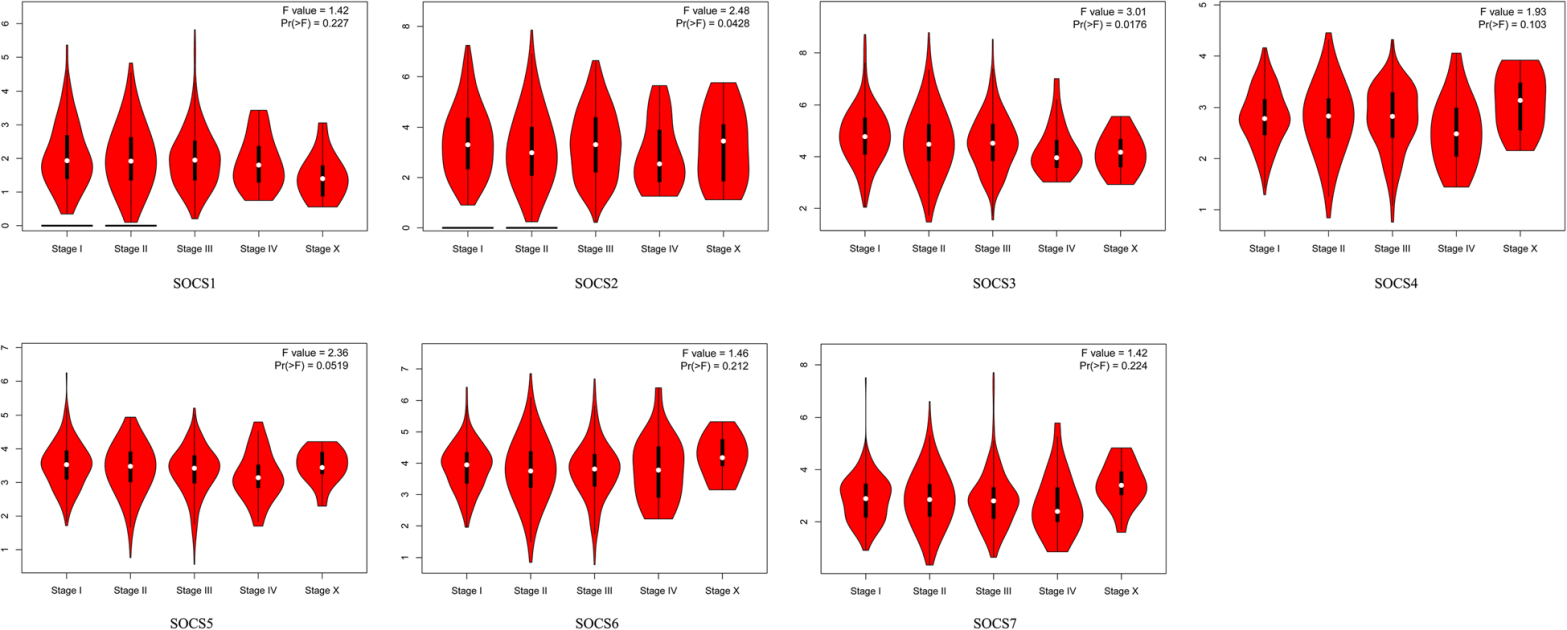
Correlation between mRNA expression levels of SOCS family members and different tumor stages in BRCA patients (GEPIA). p < 0.05 is considered statistically significant

The Kaplan-Meier plotter tool was used to analyze associations between the expression level of SOCS family members and the prognosis of BRCA patients. BRCA patients with high mRNA expression levels of SOCS3 (*p* < 0.01) and SOCS4 (*p* < 0.05) were predicted to have significantly better OS. Additionally, BRCA patients with high mRNA expression levels of SOCS2 (*p* < 0.001), SOCS3 (p < 0.001), and SOCS4 (p < 0.01) and low mRNA expression levels of SOCS5 (p < 0.001) were predicted to have better RFS (Fig. 4). Moreover, the expression of SOCS genes at different tumor stages were analyzed and the results showed that the higher the clinical stage, the lower were the expression levels of SOCS2 and SOCS3 (Fig. 5, p < 0.05). The other SOCS family members did not show any significant association with stage. Cox multivariate analyses of OS showed that SOCS3 expression level, age, and American Joint Committee on Cancer (AJCC) stage were independent prognostic factors in BRCA (Table 2). Cox multivariate analyses of RFS showed that SOCS2 expression level and AJCC stage were independent prognostic factors (Table 3).

**Table 2:** Multivariate Cox proportional hazard regression analysis of OS for patients with BRCA

Variates	HR	95% CI	P value
**SOCS3**			
**Low**	Reference		
**High**	0.668	0.438–1.019	0.048
**SOCS4**			
**Low**	Reference		
**High**	0.794	0.433–1.457	0.456
**Age**	1.031	1.014–1.048	< 0.001
**AJCC Stage**			< 0.001
**I**	Reference		
**II**	1.590	0.833–3.033	0.160
**III**	2.471	1.236–4.942	0.011
**IV**	8.158	3.421–19.459	< 0.001

AJCC: American Joint Committee on Cancer; HR: hazard ratio; CI: confidence interval

**Table 3:** Multivariate Cox proportional hazard regression analysis of RFS for patients with BRCA

Variates	HR	95% CI	P value
**SOCS2**			
**Low**	Reference		
**High**	0.680	0.487–1.095	0.034
**SOCS3**			
**Low**	Reference		
**High**	0.954	0.553–1.644	0.865
**SOCS4**			
**Low**	Reference		
**High**	0.898	0.508–1.587	0.469
**SOCS5**			
**Low**	Reference		
**High**	1.296	0.750–2.240	0.153
**Age**	0.979	0.959–1.001	0.056
**AJCC Stage**			< 0.001
**I**	Reference		
**II**	1.311	0.567–3.031	0.526
**III**	3.444	1.456–8.147	0.005
**IV**	70.265	7.471	660.835

AJCC: American Joint Committee on Cancer; HR: hazard ratio; CI: confidence interval

### Alterations, correlations and regulatory networks of SOCS family members in BRCA

**Fig. 6:**
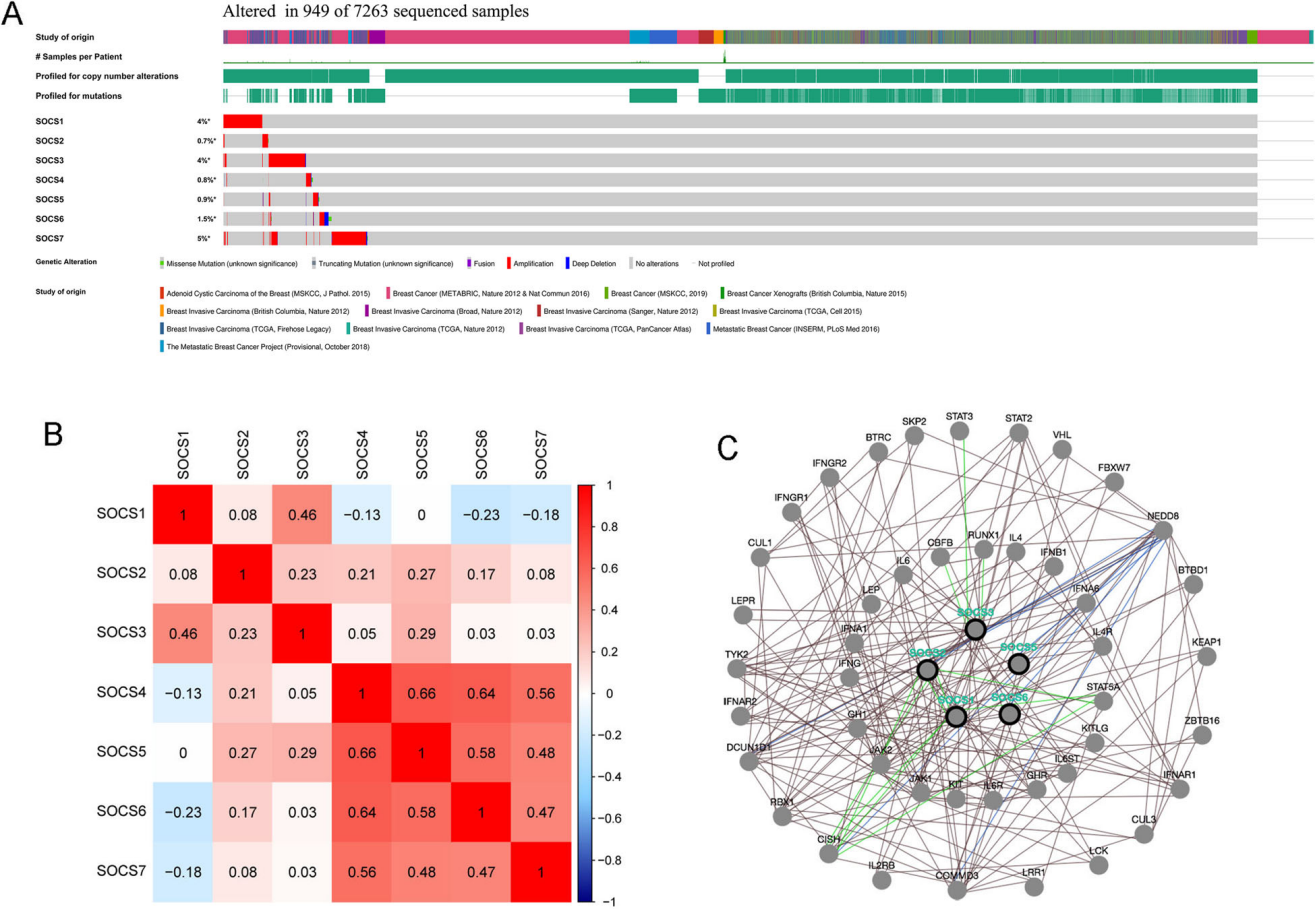
Gene expression and mutation analysis of SOCS family members in BRCA. **a** Gene expression and mutation analysis (cBioPortal). **b** Correlation between different SOCS family members in BRCA patients (TCGA). The redder the color, the stronger their positive correlation and the bluer the color, the stronger their negative correlation. **c** The regulatory networks of SOCS family members and the 50 most relevant interacting genes (PCViz). The line indicates that genes interact with each other. The more the lines, the more interactions exist

There were 1109 BRCA samples and 113 normal breast tissue samples in the TCGA database. In TCGA-BRCA RNA-seq data, the expression values of 19,597 mRNAs were identified. The alterations in SOCS genes in BRCA were analyzed using the cBioPortal online tool. As shown in Fig. 6A, SOCS genes were altered in 949 of the 7263 sequenced samples; SOCS7 mutations were the most common, followed by SOCS1 and SOCS3 mutations. Coexpression analysis was performed using the Pearson correlation method (Fig. 6B). Among the correlations, the highest correlations were observed between SOCS4 and SOCS5, 6, and 7 (|r| > 0.5 and *p* < 0.05). SOCS6 was also very highly correlated with SOCS5 (r = 0.58).

Using the PCViz tool, 327 genes and 11,112 interacting genes were identified. Since SOCS4 and SOCS7 showed no interactions with other genes, they were not included in the regulatory networks. We selected the 50 genes with the strongest interactions with each other to construct the regulatory networks associated with the SOCS family (Fig. 6C). In the interaction network, SOCS3 was found to be regulated by RUNX family transcription factor 1 (RUNX1) and signal transducer and activator of transcription 3 (STAT3), and these factors formed a complex. The expression of SOCS1 and SOCS2 was mutually regulated. SOCS1 and SOCS2 can form a complex with signal transducer and activator of transcription 5A (STAT5A), and SOCS1 and SOCS2 can thus be regulated by STAT5A. SOCS2, 3, 5, and 6 were found to regulate the change in the state of the NEDD8 ubiquitin like modifier. Cytokine-inducible SH2-containing protein regulated the expression of the SOCS1 and SOCS2 genes.

### Functional and pathway enrichment analyses of SOCS family members

**Fig. 7:**
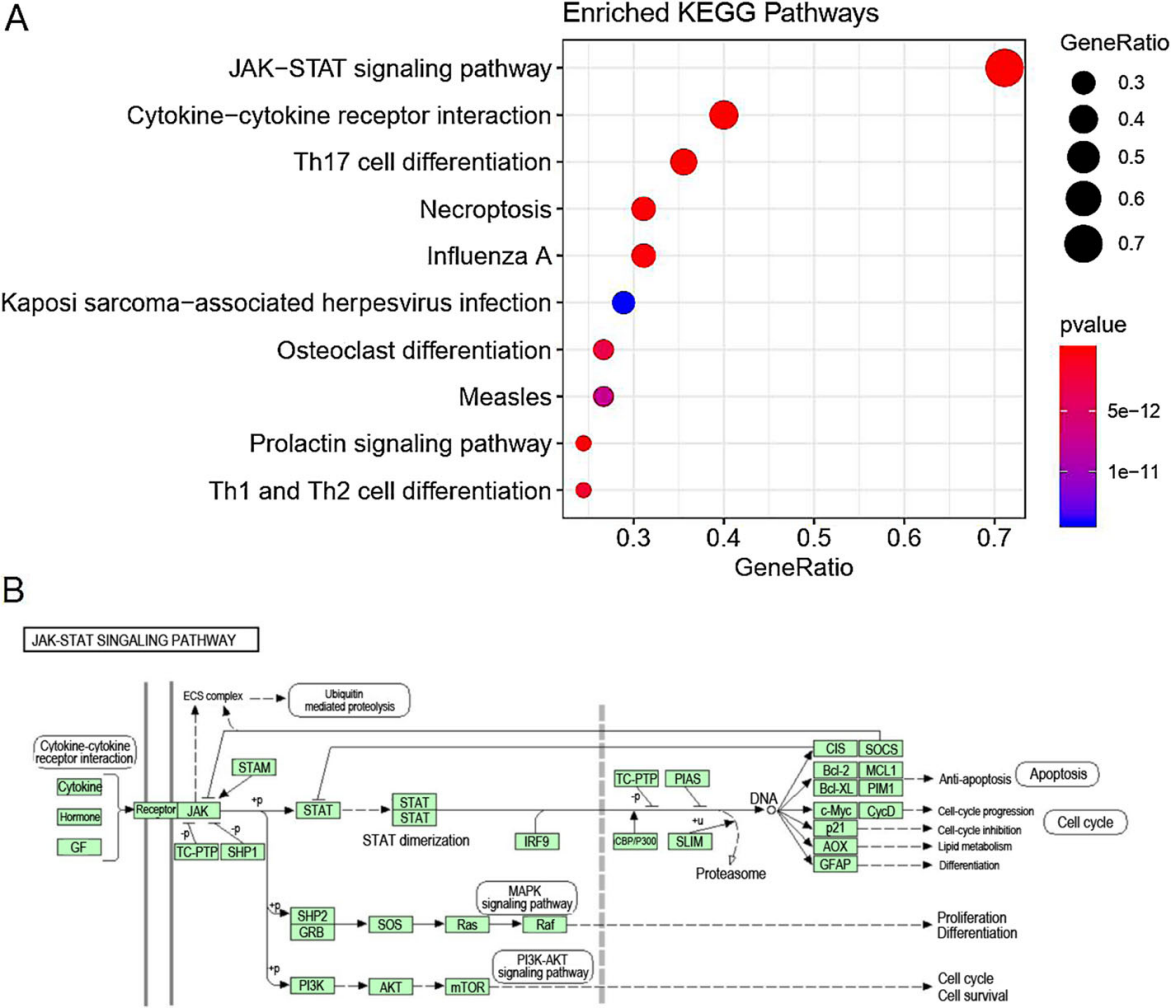
The KEGG pathways enriched for SOCS family members and the top 50 interacting genes. **a** KEGG enrichment bubble map. The dot indicates the gene cluster. The redder the color of the dots, the higher the enrichment. **b** The JAK-STAT signaling pathway regulated by SOCS family members in BRCA

**Fig. 8:**
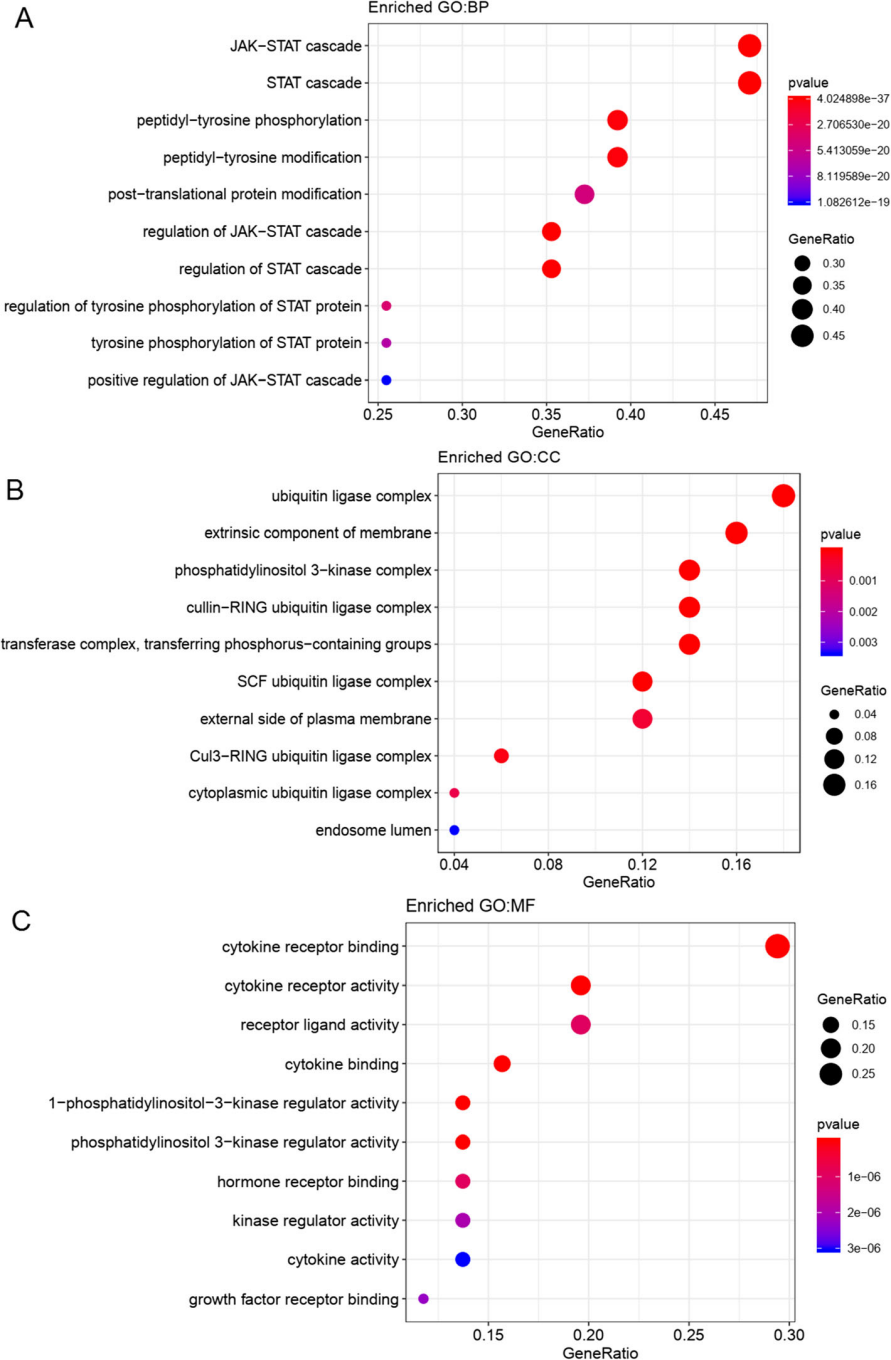
The GO functions enriched for SOCS genes and the top 50 interacting genes. **a** Biological process (BP). **b** cellular component (CC). **c** molecular function (MF). The dot indicates the gene cluster. The redder the color of the dots, the more significant the GO term

KEGG and GO enrichment analyses were performed on the top 50 genes identified in the regulatory networks, as well as on SOCS4 and SOCS7. The top three most significantly enriched KEGG pathways identified were as follows: JAK-STAT signaling, Th17 cell differentiation, and cytokine-cytokine receptor interaction (Fig. 7A). The JAK-STAT signaling pathway can be regulated by SOCS family members, as shown in Fig. 7B. The top three most significantly enriched terms in the GO BP analysis were: JAK-STAT, STAT, and regulation of JAK-STAT cascades (Fig. 8A); in the CC analysis were: phosphatidylinositol 3-kinase complex, SCF ubiquitin ligase complex, and ubiquitin ligase complex (Fig. 8B); and in the MF analysis were: cytokine receptor binding, 1-phosphatidylinositol-3-kinase regulator activity, and phosphatidylinositol 3-kinase regulator activity (Fig. 8C).

### Modules and genes related to SOCS3

**Fig. 9:**
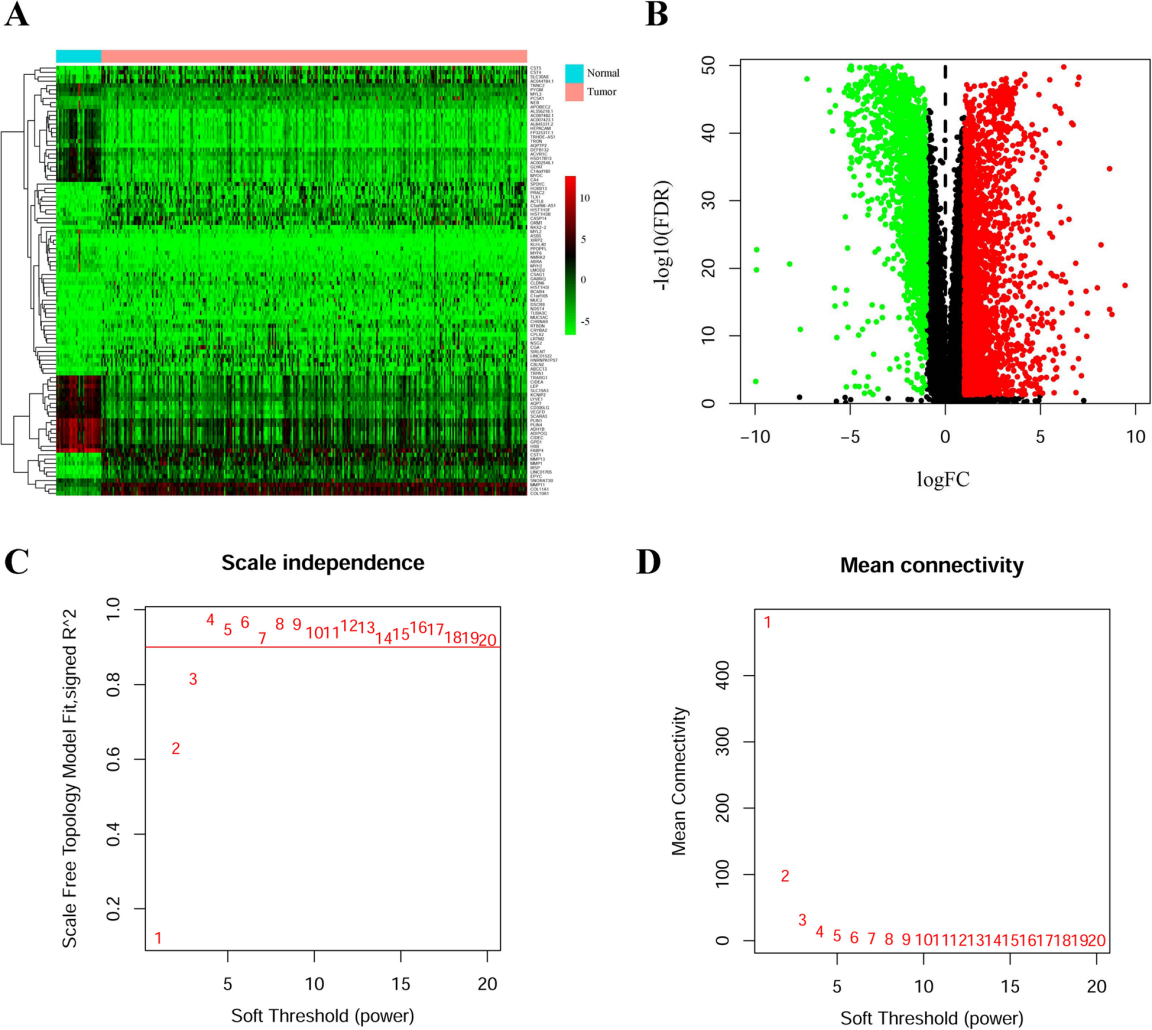
DEGs between BRCA and normal tissues in the TCGA database and Soft threshold for WGCNA. **a** Heat map. The bluer the color, the lower the gene expression and the redder the color, the higher the gene expression. **b** Volcanic plot map. Red for upregulated genes and green for downregulated genes. **c** Calculation of scale-free R^2^. **d** Analysis of mean connectivity

**Fig. 10:**
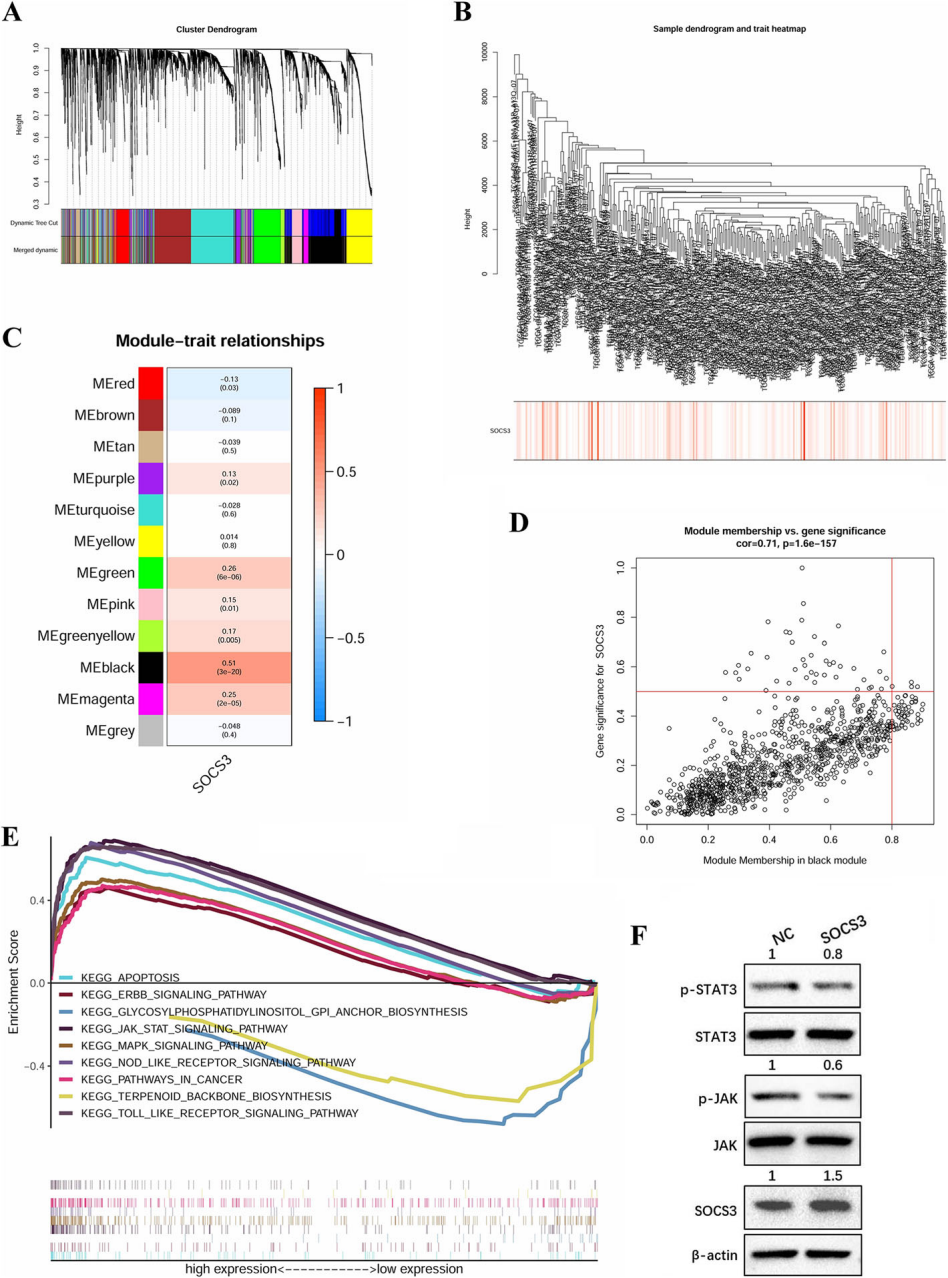
WGCNA processes and multiple GSEA. **a** Clustering dendrogram of BRCA patients. **b** DEGs clustered by the dissimilarity measure. **c** Relationship between modules and SOCS3. 12 distinct modules are generated. The darker the color, the more significant the statistical association. **d** Scatter plot of black module. **e** Enriched pathways based on GSEA. **f** Western blots showing inhibition of the JAK-STAT signaling pathway by SOCS3

Since SOCS3 was one of the most differentially expressed genes and had effects on both OS and RFS, SOCS3 was selected for further analysis in relation to BRCA. A heat map and volcano plot of differentially expressed genes (DEGs) between BRCA and normal tissues in TCGA were generated (**Fig.** **9****A** and **B**) and 4432 DEGs were identified (*p* < 0.001) and subjected to WGCNA. Scale-free R^2^ = 0.9 was selected for construction of the scale-free network (**Fig.** **9****C** and **D**). All DEGs were clustered into 12 modules; 1022 DEGs were clustered into the black module, which was most significantly associated with the SOCS3 expression level (**Fig.** **10****A-D**, r = 0.51, p < 0.001).

### The potential mechanism of SOCS3

The potential mechanism of SOCS3 was investigated by GSEA with the 1022 DEGs. The 7 upregulated KEGG pathways most significantly associated with tumor development and 2 downregulated pathways are shown in Fig. 10E**.** Notably, the JAK-STAT signaling pathway was the overlapping pathway between the pathways identified by GSEA and the KEGG pathways enriched with SOCS family members (**Fig.** **7****A** and **B**), suggesting that JAK-STAT might play a critical role in BRCA. Western blot analysis confirmed that pIRES2-SOCS2 transfection upregulated the expression of SOCS3 and reduced the levels of p-STAT3 and p-JAK in breast cancer cells (Fig. 10F**).**

### Association between SOCS3 and immune infiltration

**Fig. 11:**
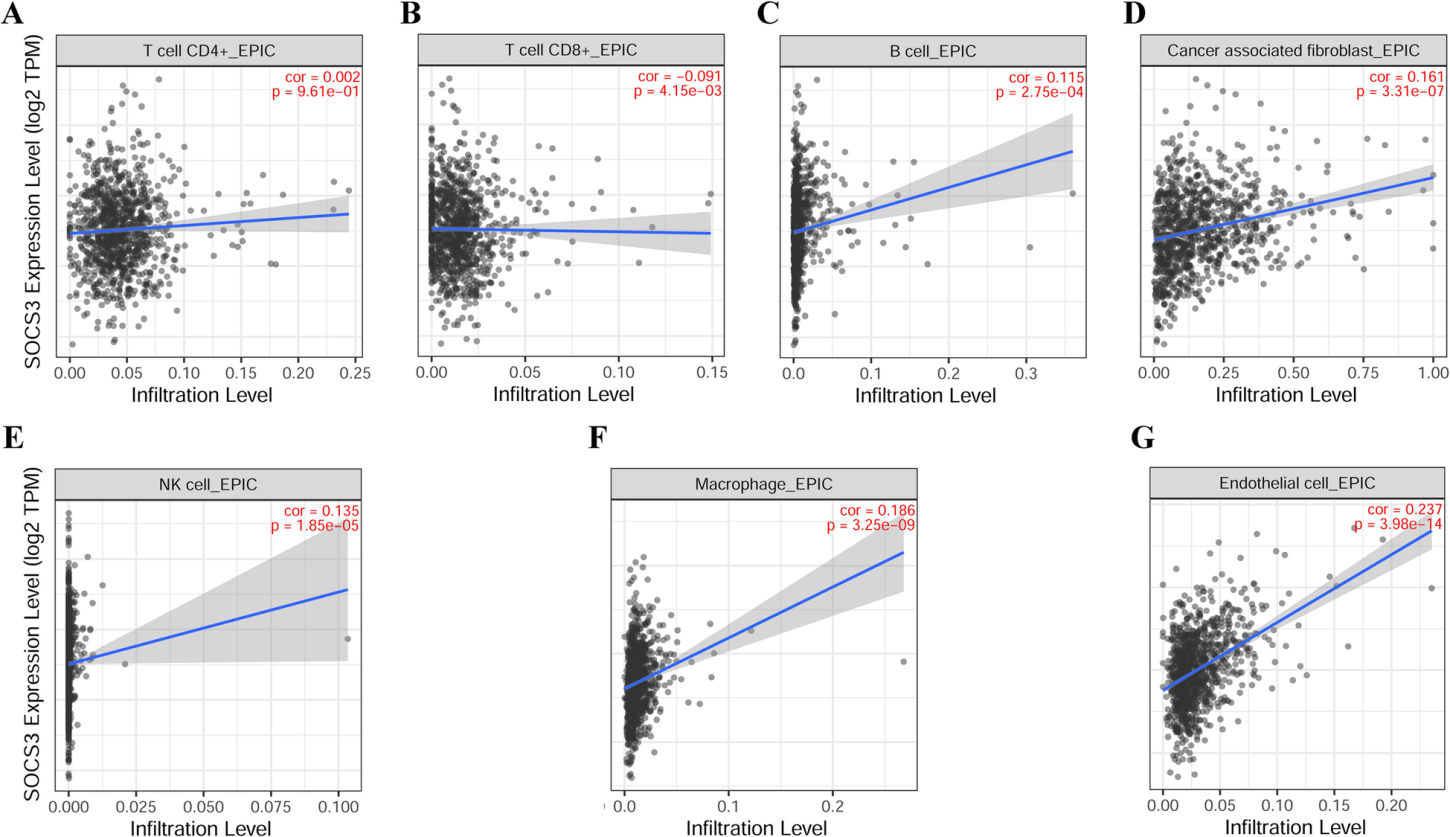
Relationship between SOCS3 and immune infiltration (TIMER). **a** SOCS3 and CD4^+^ T cells (*p* = 0.961). **b** SOCS3 and CD8^+^ T cells (*p* = 0.004). **c** SOCS3 and B cells (*p* < 0.001). **d** SOCS3 and CAFs (p < 0.001). **e** SOCS3 and NK cells (p < 0.001). **f** SOCS3 and macrophages (p < 0.001). **g** endothelial cells (p < 0.001)

TIMER was used to evaluate the association between SOCS3 and infiltrating immune cells, including CD4^+^ T cells, CD8^+^ T cells, B cells, cancer associated fibroblasts (CAFs), NK cells, macrophages and endothelial cells (Fig. 11A-G). The results showed positive correlation between the levels of SOCS3 and B cells, CAFs, NK cells, macrophages and endothelial cells. Whereas, SOCS3 and CD8^+^ T cells displayed a negative correlation with SOCS3 levels in BRCA.

### Associations between SOCS3 and methylation, copy number and clinical parameters

**Fig. 12:**
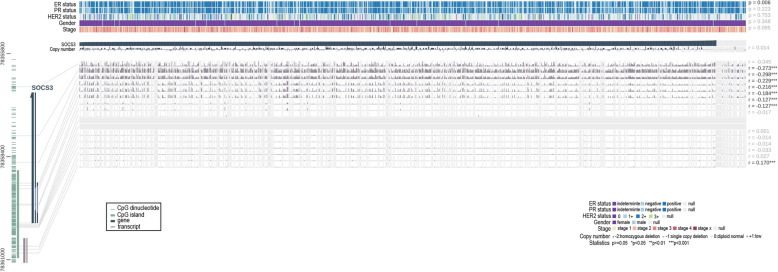
Association between SOCS3 and methylation, copy number and clinical parameters (MEXPRESS). p < 0.05 is considered statistically significant

MEXPRESS was used to explore the association between SOCS3 expression and methylation and found that methylation at CpG locations 78,360,830, 78,357,871, 78,358,540, 78,359,065, 78,359,121, 78,359,208, 78,359,594 and 78,359,620 exhibited a statistically significant relationship with SOCS3 expression (Fig. 12). No statistically significant association was detected between SOCS3 expression and copy number alteration. With respect to common clinical parameters including ER status, PR status, HER2 status, gender and stage, SOCS3 was found to be significantly correlated only with ER status (*p* < 0.01).

## Discussion

Breast cancer is still a considerable challenge due to its high rates of recurrence and metastasis [28]. SOCS genes and their interacting genes were found to be significantly involved in the JAK-STAT signaling pathway. Numerous researchers have verified the important roles of SOCS family members in malignant processes [8, 29]. SOCS family members also play vital roles in regulating antitumour immunity [30, 31]. To the best of our knowledge, our study is the first to investigate the mRNA expression levels and prognostic value of SOCS family members in BRCA. We found that SOCS2 and SOCS3 had lower expression levels in BRCA tissues than in normal breast tissues. Higher mRNA expression levels of SOCS3 and SOCS4 were significantly associated with longer OS times. Multivariate analyses showed that SOCS3 was an independent prognostic factor for OS. Higher levels of SOCS2, 3, 4, and 5 were significantly associated with longer RFS times. Multivariate analyses showed that SOCS2 was an independent prognostic factor for RFS. Therefore, our results may contribute to improving the treatment and enhancing the prognostic accuracy for patients with breast cancer.

SOCS2 belongs to the family of ubiquitin ligases, and is a target recognition subunit of an E3 ubiquitin ligase complex [32]. Deletion of SOCS2 promotes spontaneous development of intestinal tumors in mice [33]. Furthermore, the expression of SOCS2 is lower in BRCA tissues than in normal control tissues, and thus, high SOCS2 expression is associated with good prognosis [34, 35]. Consistent with these findings, our study also showed low expression of SOCS2 in BRCA patients, and a high mRNA level of SOCS2 was predicted to be associated with more favorable RFS. Interestingly, the regulatory networks of SOCS genes showed that SOCS2 was regulated by STAT5A. STAT5A was first identified as a mammary gland factor [36]. Phosphorylated STAT5A has been shown to regulate the expression of target genes promoting the survival, proliferation and differentiation of breast epithelial cells through nuclear translocation, functional dimerization and DNA binding [37]. Activated STAT5A is known to promote the differentiation and suppress the invasive features of breast cancer cells [38]. Therefore, we speculated that low expression of SOCS2 may promote breast cancer progression through regulation of STAT5A.

Increasing evidence has suggested a role of SOCS3 in breast cancer as a regulator of STATs [39]. Overexpression of SOCS3 has been associated with an enhanced antiproliferative effect [39]. SOCS3 expression is decreased in breast cancer tissues compared with normal tissues, and a lower level of SOCS3 is associated with poor clinical outcomes [40–42]. In our study, SOCS3 had lower expression levels in tumor tissues than in normal tissues. In addition, a high mRNA expression level of SOCS3 was predicted to be associated with a longer OS time, consistent with the above-mentioned findings. However, Raccurt et al. [43] reported that SOCS3 is overexpressed in breast ductal carcinoma tissue compared with adjacent normal tissue. Therefore, the precise roles of SOCS3 in breast cancer are still controversial, and this inconsistency in the findings may be due to the heterogeneity of breast cancer tissues. Tumor development is related to various inflammatory signaling pathways, including the JAK-STAT signaling pathway [44]. Aberrant JAK2/STAT3 signaling has been reported in various types of tumors, including breast cancer [45, 46]. SOCS3 is a negative feedback regulator of STAT3, and thus can suppress the STAT3 signaling pathway [47]. STAT3 is aberrantly activated in approximately 70% of breast cancer patients [48]. Kajari et al. [49] showed that the STAT3-NFkBp65 interaction caused promoter hypermethylation of SOCS3, which subsequently led to downregulation of SOCS3. Consistent with the above findings, in our study, the results of regulatory networks and pathway analyses also revealed that SOCS3 had a significant negative correlation with the level of methylation, and was involved in the JAK-STAT signaling pathway. Collectively, these findings indicate that a decrease in the expression of SOCS3 may promote the progression of breast cancer via the JAK-STAT signaling pathway and thereby worsen its prognosis.

Less is known about the roles of SOCS4 than about the roles of SOCS2 and SOCS3 in tumor growth and malignancy. Kobayashi et al. [50] reported downregulation of SOCS4 in gastric cancer tissues compared with normal tissues and showed that hypermethylation of SOCS4 was associated with poor prognosis. A recent study also reported that the expression of SOCS4 was decreased in thyroid cancer cells [51]. Sasi et al. [13] found that high expression of SOCS4 was correlated with early-stage tumor, which was further associated with more favorable OS and a marginal benefit to RFS in breast cancer patients. In our study, high expression of SOCS4 was also associated with longer OS and RFS times in breast cancer patients.

Yoon et al. [52] investigated the expression patterns of SOCS5 and SOCS6 in many human cancer and normal tissues using a Cancer Profiling Array and found that these two genes exhibited similar expression levels in patients with most cancer types and healthy individuals, indicating that SOCS5 and SOCS6 are transcriptionally coregulated. These results were consistent with our findings that SOCS6 expression was significantly and positively correlated with SOCS5 expression. Sasi et al. [13] reported that SOCS5 was significantly downregulated in BRCA tissues compared with normal tissues. Decreased expression of SOCS5 was correlated with advanced tumor, but no significant correlation was found between decreased SOCS5 expression and improved RFS or OS. In our study, SOCS5 was downregulated in tumor tissues, and there was no indication of a significant association between low expression of SOCS5 and improved OS, consistent with the results of Sasi et al. [13]. As a tumor suppressor, SOCS6 was shown to inhibit cell proliferation in breast cancer [53] and was usually suppressed in tumor cells such as gastric, liver and prostate tumor cells [54–56]. In our study, however, we revealed that SOCS6 was upregulated in BRCA tumor tissues, which may also be the case in different cancer types. Therefore, further studies are still required to confirm the role of SOCS6 in breast cancer.

We did not identify significant differences in the expression of SOCS1 or SOCS7 between cancer and normal samples. Sasi et al. [13] reported that high expression of SOCS1 and SOCS7 was associated with early-stage tumor and more favourable prognosis in patients with breast cancer. However, the findings regarding the role of SOCS1 in BRCA in different studies are inconsistent. For instance, Qian et al. [57] reported that SOCS1 was overexpressed in triple-negative breast cancer tissues and cell lines compared with normal mammary tissues and cell lines. Conversely, Lv et al. [6] showed that SOCS1 had lower mRNA expression levels in breast cancer tissues than in adjacent normal tissues. These inconsistencies may be attributed to the heterogeneity of breast cancer tissues.

## Conclusion

In conclusion, SOCS family members play a very important role in BRCA. SOCS3 may be a prognostic factor, and SOCS2 may be a potential therapeutic target in breast cancer. In addition, our present data validated and supported bioinformatic analysis as an appropriate starting point for analyzing breast cancer and discovering novel biomarkers and therapeutic targets for breast cancer.

## Supplementary Information

**Additional file 1.** Original western blotting band pictures.

## Data Availability

The datasets generated and analyzed during the current study are available from the corresponding author on reasonable request. Web sources: TCGA (http://tcga-data.nci.nih.gov/tcga/); Oncomine (http://www.oncomine.org); GEPIA (http://gepia.cancer-pku.cn/); GTEx (http://commonfund.nih.gov/GTEx/); Kaplan-Meier Plotter (http://www.kmplot.com); cBioPortal (http://www.cbioportal.org); HPA (http://www.proteinatlas.org/); TIMER 2.0 (http://timer.cistrome.org/); MEXPRESS (http://mexpress.be).

## Abbreviations

SOCS: Suppressor of cytokine signaling
CIS: cytokine-inducible SH2-containing protein
TCGA: The Cancer Genome Atlas
GTEx: Genotype-Tissue Expression
BRCA: breast invasive carcinoma
GEPIA: Gene Expression Profiling Interactive Analysis
OS: overall survival
RFS: recurrence-free survival
HPA: The Human Protein Atlas
KEGG: Kyoto Encyclopedia of Genes and Genomes
GO: Gene Ontology
BP: biological process
MF: molecular function
CC: cellular component
WGCNA: Weighted gene coexpression network analysis
GSEA: Gene set enrichment analysis
RUNX1: RUNX family transcription factor 1
STAT3: signal transducer and activator of transcription 3
STAT5A: signal transducer and activator of transcription 5A
DEGs: differentially expressed genes
CAF: cancer associated fibroblast
AJCC: American Joint Committee on Cancer
HR: hazard ratio
CI: confidence interval
