# Supplementary material for: Comprehensive analysis of suppressor of cytokine signaling proteins in human breast Cancer
Source: BMC Cancer. 2021 Jun 13;21:696. doi: 10.1186/s12885-021-08434-y (PMC8201682; doi:10.1186/s12885-021-08434-y)

Supplementary Information file 1. **Original western blotting band pictures.**

β-actin


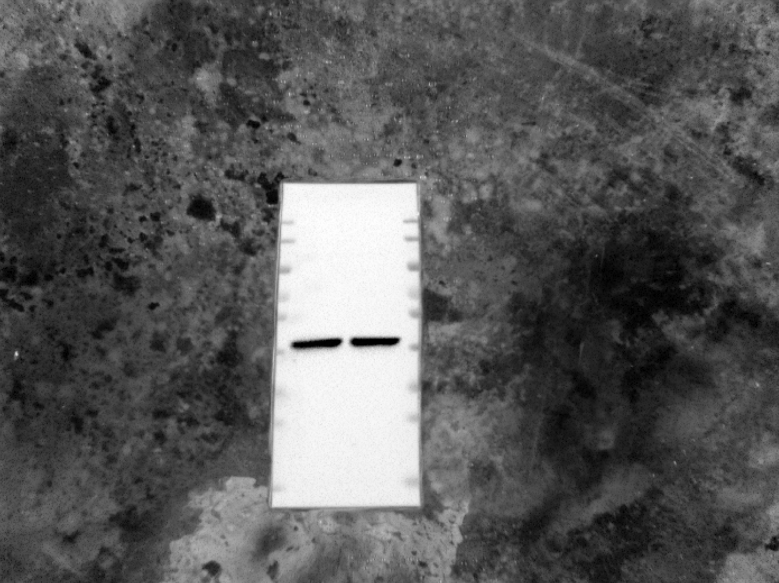


SOCS3


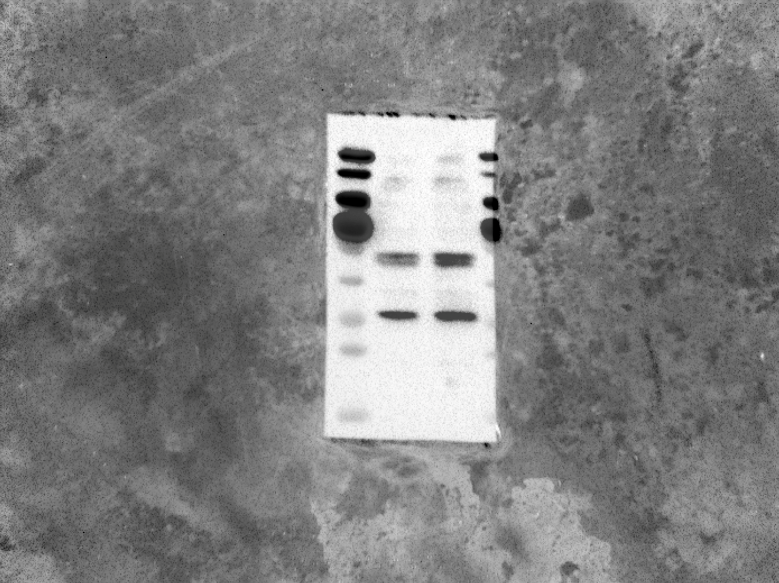


JAK


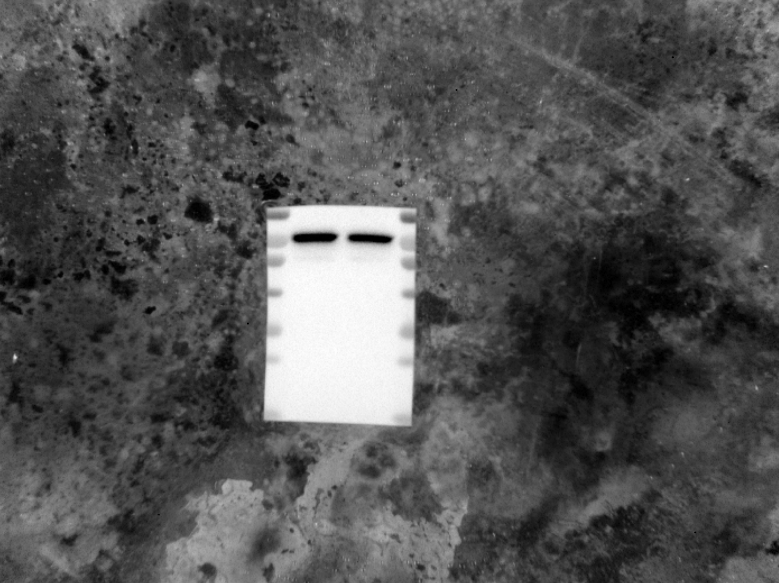


p-JAK


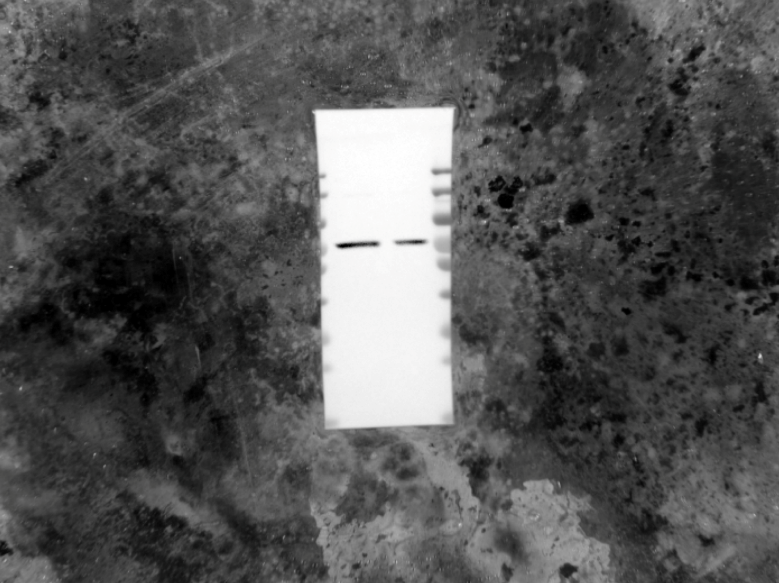


STAT3


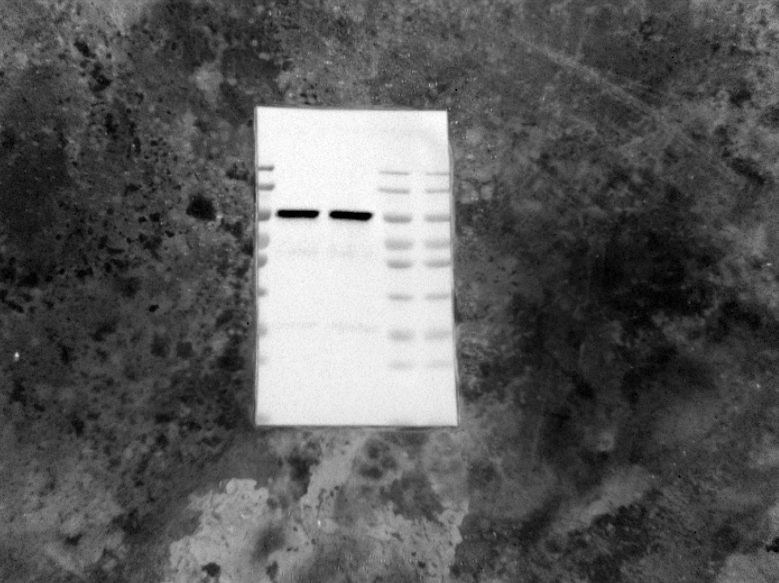


p-STAT3


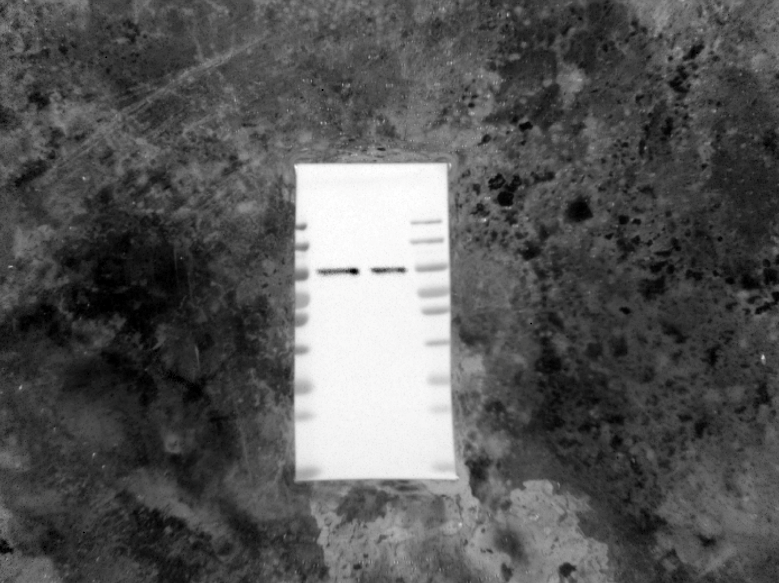

Supplement: Supplementary file 1 — Additional file 1. Original western blotting band pictures. [file 12885_2021_8434_MOESM1_ESM.docx]
